# Seizure as Presenting Symptom of Multisystem Inflammatory Syndrome in Children

**DOI:** 10.1155/2023/3581310

**Published:** 2023-07-08

**Authors:** Eleonora S. D'Ambrosio, Stefanie Gauguet, Christine Miller, Erin McMahon, Christopher Driscoll, Mugdha Mohanty, Thomas Guggina

**Affiliations:** ^1^Department of Neurology, University of Massachusetts Medical Center, University of Massachusetts Chan Medical School, 55 Lake Avenue North, Worcester, MA 01655, USA; ^2^Department of Pediatrics, UMass Memorial Children's Medical Center, University of Massachusetts Chan Medical School, 55 Lake Avenue North, Worcester, MA 01655, USA

## Abstract

We describe the case of a 13-year-old girl who presented with a new-onset seizure and fever and subsequently developed severe cardiac dysfunction, coronary artery dilation, and shock due to the surprising diagnosis of multisystem inflammatory syndrome in children (MIS-C). Although the clinical entity we now call MIS-C was first mentioned in the medical literature in April 2020, the full picture of this disease process is still evolving. Neurologic involvement has been described in cases with MIS-C; however, seizures are not a typical presenting symptom. Additionally, because children infected with SARS-CoV-2 are often asymptomatic, a documented preceding COVID-19 infection might not be available to raise suspicion of MIS-C early on. Febrile seizures, meningitis, and encephalitis are childhood illnesses that pediatricians are generally familiar with, but associating these clinical pictures with MIS-C is uncommon. Given the possibility of rapid clinical cardiogenic decline, as seen in our patient, a prompt diagnosis and appropriate monitoring and treatment are of utmost importance. This case report aims to raise awareness that new-onset seizures with fevers can be early or the first presenting symptoms in children with MIS-C, and further workup and close monitoring may be required.

## 1. Case Presentation

An overweight but otherwise healthy 13-year-old African-American girl presented to the emergency department (ED) with a suspected first-time seizure. Her parents called 911 after finding her unresponsive and rigidly lying on the floor. There was no associated pallor, cyanosis, or incontinence. The rigidity lasted for approximately five minutes and was followed by a period of confusion. This was preceded by three days of occipital headache, neck pain, fatigue, diffuse abdominal pain, diarrhea, one day of an itchy, raised rash on her legs and arms, and a mild conjunctival injection. There was no reported trauma, suspected ingestion, or illness in the preceding month. The patient had no recollection of this event. She reported no loss of sense of taste or smell at any point in time and had neither undergone any recent coronavirus disease 2019 (COVID-19) testing nor previous vaccination against SARS-CoV-2. She had been afebrile until her presentation in the ED.

In the ED, she was febrile to 39.5°C, had a pulse of 138, respiratory rate of 25, a blood pressure 116/58 mm·Hg, and a weight of 62.5 kg. She appeared generally well, complained of pain with neck movement, and had full range of motion. Heart, lung, abdominal, and neurologic exams were unremarkable. She had a raised, blanching, erythematous rash on her forearms and thighs. Lab results were significant for lymphopenia, normocytic anemia, mild hyponatremia, low bicarbonate, and slightly elevated glucose. Her LFTs and albumin were normal on admission. A blood culture was obtained and remained sterile (Table 1). A lumbar puncture (LP) was attempted unsuccessfully. She was admitted for observation with suspicion for viral versus Lyme meningitis. Brain imaging studies were deferred at the time of the initial evaluation, considering that her mental status and neurological exam were reassuringly normal. A total of two one-liter normal saline boluses were administered in the ED for tachycardia and presumed dehydration.

The following morning, an LP was repeated successfully. The opening pressure was elevated to 31 cm H_2_O, but CSF cell counts were reassuring. CSF was sent for cultures, Gram stain, and a meningitis-encephalitis panel, which were all negative. A mini-RVP panel from a nasopharyngeal swab (which included COVID-19, Influenza A and B, RSV, and hMPV) was negative. Repeat metabolic studies and inflammatory markers were obtained as well, and due to persistent fevers, SARS-CoV-2 IgG levels were sent. Over the next 24 hours the patient's fevers remained high, and she became progressively more tachycardic. Early on hospital day 2 she developed pallor, hypotension (65/32 mm·Hg), cool extremities, thready pulses, and a new S3 gallop on exam, concerning for hypotensive shock. She was immediately transferred to the pediatric intensive care unit (PICU), given crystalloid boluses, and started on a norepinephrine infusion. Around this time, her SARS-CoV-2 IgG levels were reported as positive, and her inflammatory markers were highly elevated, raising suspicion for multisystem inflammatory syndrome in children (MIS-C).

An echocardiogram showed a reduced left ventricular ejection fraction (LVEF) to 44% as well as left anterior descending coronary artery dilation and ectasia. Treatment for MIS-C including methylprednisolone (2 mg/kg/day IV), intravenous immunoglobulin (IVIG) (2 g/kg IV), and aspirin (325 mg) by mouth daily was initiated. Over the course of the evening, she developed acute respiratory failure with hypoxia requiring noninvasive mechanical ventilation in the form of high-flow nasal cannula oxygen therapy initially, escalating to full facemask bilevel positive airway pressure (BiPAP) within a few hours. A chest radiograph obtained at this time showed worsening pulmonary edema as well as a developing left lower lobe pneumonia. A repeat echocardiogram showed a further reduction in her LVEF to 43% as well as mild left coronary ectasia. She was started on ceftriaxone and azithromycin to treat the pneumonia and furosemide to treat her pulmonary edema. Enoxaparin and anakinra (300 mg twice daily IV) were initiated as well.

After 18 hours of inotropic support, her blood pressure stabilized and norepinephrine was discontinued. During hospital days 3-4, our patient had several abnormal findings on telemetry and electrocardiogram, including dropped P-waves and prolonged PR intervals concerning a 1^st^ degree heart block. A repeat echocardiogram showed improvement of her cardiac function with her LVEF at 52% along with slightly improved coronary artery size and resolution of the ectasia that had been seen previously. An electroencephalogram was obtained which was normal. Her albumin levels, which were normal on admission, had also decreased to 2.3 g/dL. By hospital day 4, the patient was showing signs of stabilization and requiring less respiratory support. By day 6, she was weaned to room air, anakinra was stopped, and she was transferred back to the pediatric floor where she continued to regain strength and diurese. A brain MRI obtained on hospital day 8 was reassuringly normal except for a small T2 hyperintensity in the genu of the corpus callosum. She was discharged home on hospital day 9 in good health, on aspirin and a steroid taper. At follow-up 4.5 months later, she had a normal ECHO, a normal neurological appearance, was symptom-free, and overall felt well.

## 2. Discussion

Around April 2020, it was recognized that children are generally spared the acute respiratory manifestations of COVID-19 but are susceptible to the potentially serious and life-threatening postinfectious Kawasaki-like, multisystem inflammatory syndrome MIS-C [1, 2]. Patients with MIS-C often present with several days of fever, multiple organ system involvement, most commonly gastrointestinal, mucocutaneous, and cardiovascular, and no other explanation for their symptoms [1–3].

Neurologic symptoms had been described in a minority of patients with MIS-C [4] early on but are recognized to be more common than initially thought [5]. While most of these patients have headaches as their main neurologic symptom, seizures, altered mental status, and coma have also been reported.

Coronaviruses have a long history of a broad range of neurologic manifestations including serious neurologic disease processes such as acute flaccid paralysis, acute disseminated encephalomyelitis, seizures, encephalitis, and stroke [6]. Numerous hypotheses may explain the effect of the SARS-CoV-2 virus on the nervous system, including direct effect of the virus, endothelial damage, and para- and postinfectious inflammatory cytokine cascades causing nerve damage and demyelination [7].

Seizures have been reported in cases of COVID-19 and MIS-C [8, 9], but generally not as the presenting symptom. In this case, our patient presented with an apparent seizure and had her first measured fever in the emergency department. The lack of prolonged fever and absence of other well-known symptoms of acute COVID-19 infection did not raise suspicion for MIS-C initially. The patient underwent a workup for a first-time seizure and fever in a standard manner, by detailed clinical assessment and laboratory evaluation to identify a provoked cause for the seizure. Given the persistent fever and meningismus, a lumbar puncture was performed, and once the patient was stabilized neuroimaging was obtained, helping to identify other causes of seizures.

While it is possible that the initial event may have represented a syncopal episode, the deviated gaze, prolonged stiff posturing, tongue protrusion, amnesia, and apparent postictal period would suggest this was a seizure. Additionally, our patient had elevated opening pressure on lumbar puncture, which has been reported in other cases of MIS-C [10] and further implicates involvement of the central nervous system.

Though older than the typical patient with a simple febrile seizure in the pediatric population, our patient's general appearance and the presence of a rash which appeared consistent with a viral infection were reassuring enough that it was reasonable to admit her for observation without antibiotics. As fevers persisted and meningitis, encephalitis, and sepsis were ruled out, the differential diagnosis was expanded to include MIS-C. The results of positive SARS-CoV-2 IgG and highly elevated inflammatory markers coincided with her abrupt hemodynamic decline, and the diagnosis of MIS-C became suddenly clearer. Fortunately, our patient responded well to careful fluid management, vasopressors, and respiratory support and has experienced no long-term sequelae to this point.

Although she has recovered seemingly fully from her MIS-C-related cardiac involvement and no adverse event occurred, we are aware that our patient's clinical course could have easily deteriorated and led to a catastrophic outcome. Had we been more attuned to the possibility of MIS-C earlier in her course, perhaps we could have prevented or ameliorated her hemodynamic deterioration with earlier initiation of steroids and IVIG [11]. We, therefore, want to raise awareness of the possibility of MIS-C as an underlying condition in a child presenting with new seizures and its potentially rapidly evolving clinical course.

With febrile seizures and aseptic meningitides being relatively common conditions among children, it will be critical for the physicians caring for pediatric patients to be aware that MIS-C should also be considered when evaluating children with seizures and fever. Our case demonstrates the need to expand the index of suspicion for MIS-C in patients who present with acute neurologic symptoms, such as first-time seizure and fever, even if the fever is new in onset and no preceding COVID-19 disease is reported.

## Figures and Tables

**Table 1:** Laboratory values.

	Hospital day 0	Hospital day 2	Hospital day 3	Hospital day 4	Hospital day 9	Follow-up visit 1 month after discharge
WBC (4.5–13.0 × 10 *∗* 3/*μ*L)	**15.8**	**20.7**	**20.3**	**28**	10.7	4.7
WBC differential	Automated	Automated			Manual	Automated
Neutrophils (%)	**91**	**92**			77	39.1
Bands		17			13	
Lymphocytes (%)	**2.4**	**1.9/1**			14	50.2
Monocytes (%)	**6.3**	**4.4/1**			5	8.2
Eosinophils (%)	**0.2**	**1.6/2**			0	0.9
Basophils (%)	**0.1**	**0.1**/−			0	1.6
Hemoglobin (11.5–15.3 g/dL)	9.6	8.3	13.4	7.1	11.4	12.0
Platelets (140–440 × 10 *∗* 3/*μ*L)	185	154	**130**	210	273	293
Albumin (3.2–5 g/dL)	3.4	**3.0**	**2.3**	**2.7**	**2.8**	4.0
AST (12–78 U/L)	11	13		7	10	12
ALT (15–37 U/L)	25	20		16	22	9
Bilirubin, total (0.3–1.2 mg/dL)	1.1	0.9		0.4	0.2	0.3
Blood glucose (70–99 mg/dL)	**108**	**113**	**107**	**137**	**109**	90
Sodium (135–145 mmol/L)	**129**	138	135	140	138	136
Potassium (3.5–5.3 mmol/L)	3.6	**3.1**	**3.3**	3.5	4.4	4
Chloride (97–110 mmol/L)	**96**	106	106	107	102	107
Calcium (8.7–12.7 mg/dL)	8.7	**8.6**	**7.9**	**8.2**	**8.2**	9
Magnesium (1.6–2.4 mg/dL)	2.2				2.1	
Phosphate (3.5–5.5 mg/dL)	2.0				3.0	
ESR (<20 mm/Hr)		**100**				15
CRP (<10.0 mg/L)		**>400.0**	**350.1**	**183.5**	**15.3**	1.4
Troponin (0.01–0.04 ng/mL)		0.05	**1.86**	**0.4**	**0.16**	<0.015
BNP (<100 pg/mL)		**900**	**1310**	**2159**	**118**	5
D-dimer (<0.50 mg/L FEU)		**2.66**	**3.53**	**2.42**	**3.92**	**0.60**
IL-6 (<2.0 pg/mL)		**30.5**				
Ferritin (11.0–306.0 ng/mL)		**541**				
Fibrinogen (150–440 mg/dL)		**687**	**654**	**558**	**237**	
SARS-CoV-2 IgG		**Positive**				
SARS-CoV-2 PCR		Negative				
Creatinine (0.4–1.0 mg/dL)	0.82	1.6	0.67	0.7	0.45	0.56
WBC, CSF (cell/mm^3^)		1				
RBC, CSF (cell/mm^3^)		147				
Glucose, CSF (50–80 mg/dL)		76				
Protein, CSF (15–45 mg/dL)		26				
Blood culture	Negative					
CSF culture		Negative				

Abnormal values are indicated in bold.

## Data Availability

The data used to support the findings of this study are included within the article.

## Abbreviations

ED: Emergency department
LP: Lumbar puncture
COVID-19: Illness caused by SARS-CoV-2; coronavirus disease 2019
SARS-CoV-2: Severe acute respiratory syndrome coronavirus 2
LFT: Liver function test
RVP: Respiratory viral panel
RSV: Respiratory syncytial virus
hMPV: Human metapneumovirus
IVIG: Immunoglobulin G
PICU: Pediatric intensive care unit
MIS-C: Multisystem inflammatory syndrome in children
BiPAP: Bilevel positive airway pressure
LVEF: Left ventricular ejection fraction
WBC: White blood count
AST: Aspartate aminotransferase
ALT: Alanine aminotransferase
ESR: Erythrocyte sedimentation rate
CRP: C-reactive protein
BNP: B-type natriuretic peptide
IL-6: Interleukin 6 protein
IgG: Immunoglobulin G
PCR: Polymerase chain reaction
CSF: Cerebrospinal fluid.
